# Reliability and concurrent validity of the Dutch hip and knee replacement expectations surveys

**DOI:** 10.1186/1471-2474-11-242

**Published:** 2010-10-19

**Authors:** Inge van den Akker-Scheek, Jos JAM van Raay, Inge HF Reininga, Sjoerd K Bulstra, Wiebren Zijlstra, Martin Stevens

**Affiliations:** 1 Department of Orthopedics, Martini Hospital Groningen, Groningen, The Netherlands; 2 Department of Orthopedics, University Medical Center Groningen, University of Groningen, PO Box 30.001, 9700 RB, Groningen, The Netherlands; 3 Center for Human Movement Sciences, University Medical Center Groningen, University of Groningen, Groningen, The Netherlands

## Abstract

**Background:**

Preoperative expectations of outcome of total hip and knee arthroplasty are important determinants of patients' satisfaction and functional outcome. Aims of the study were (1) to translate the Hospital for Special Surgery Hip Replacement Expectations Survey and Knee Replacement Expectations Survey into Dutch and (2) to study test-retest reliability and concurrent validity.

**Methods:**

Patients scheduled for total hip (N = 112) or knee replacement (N = 101) were sent the Dutch Expectations Surveys twice with a 2 week interval to determine test-retest reliability. To determine concurrent validity, the Expectation WOMAC was sent.

**Results:**

The results for the Dutch Hip Replacement Expectations Survey revealed good test-retest reliability (ICC 0.87), no bias and good internal consistency (alpha 0.86) (N = 72). The correlation between the Hip Expectations Score and the Expectation WOMAC score was 0.59 (N = 86). The results for the Dutch Knee Replacement Expectations Survey revealed good test-retest reliability (ICC 0.79), no bias and good internal consistency (alpha 0.91) (N = 46). The correlation with the Expectation WOMAC score was 0.52 (N = 57).

**Conclusions:**

Both Dutch Expectations Surveys are reliable instruments to determine patients' expectations before total hip or knee arthroplasty. As for concurrent validity, the correlation between both surveys and the Expectation WOMAC was moderate confirming that the same construct was determined. However, patients scored systematically lower on the Expectation WOMAC compared to the Dutch Expectation Surveys. Research on patients' expectations before total hip and knee replacement has only been performed in a limited amount of countries. With the Dutch Expectations Surveys it is now possible to determine patients' expectations in another culture and healthcare setting.

## Background

Osteoarthrosis is the most common joint disorder in the world [1]. Patients with osteoarthrosis of the hip or knee joint experience pain, stiffness and loss of joint function. When conservative treatment does not result in less pain and better functioning, a total hip or knee replacement is the most common and successful surgical treatment. In 2005, the incidence of hip replacements in the Netherlands was 124 per 10^5 ^inhabitants (20,281 operations); the incidence of knee replacements was 63 per 10^5 ^inhabitants (10,329 operations) (Statistics Netherlands 2005).

In orthopaedics, increasing emphasis is placed on patient-reported outcome of the surgery, patient satisfaction and quality of life, and not solely on the technical success of the surgical procedure and the surgeon's rating of the outcome. Previous research has indicated that preoperative expectations are important determinants of patients' satisfaction and functional outcome of total joint replacement [2-6]. Patients have multiple expectations of the outcome of total hip or knee replacement, mainly concerning relief of pain and improvement in physical function and psychosocial well-being [3]. Fulfilled expectations are linked to increased patient compliance with postoperative recommendations and return to follow-up care and monitoring [7]. Unrealistically high expectations can result in discouraged patients postoperatively and non-adherence with recommendations postoperatively, while unrealistically low expectations can result in less motivation to obtain full benefit from the surgery [7].

Additionally, research indicates that differences exist between the ratings of patients and surgeons on the outcome of the surgery [8]. Among the many explanations for this difference, it is hypothesised that a difference in expectations plays an important role [9]. It is therefore important to assess patients' expectations before surgery. Especially patients with a poor preoperative status often have high expectations which are potentially unrealistic [10].

To avoid unrealistic expectations, it has been recommended to query patients about their expectations before surgery [3,11]. Physician-patient discussions about preoperative expectations should be an important part of clinical care [12]. Moreover, preoperative education classes have shown the ability to change the expectations and result in more equal expectations of patient and surgeon [13,14]. Questionnaires can be used to guide discussions and evaluate interventions. However, no Dutch questionnaires are available to determine patients' expectations before total hip or knee arthroplasty. Therefore, the aims of the current research were (1) to translate the English-language Hospital for Special Surgery Hip Replacement Expectations Survey [3,10,13] and the Hospital for Special Surgery Knee Replacement Expectations Survey [13,15] into Dutch according to international guidelines as described by Beaton et al. [16], and (2) to study test-retest reliability and concurrent validity of the two Dutch-language surveys.

## Methods

### Questionnaires

The Hospital for Special Surgery Hip Replacement Expectations Survey is developed by Mancuso et al. to determine patient expectations before the surgery [3,10]. By means of interviews with 180 patients about their expectations and reviews of these patient-derived items by a panel of orthopaedic surgeons, eventually 18 items were included in the self-report questionnaire. Expectations related to symptoms, physical activity, work and psychological well-being were assessed. Patients were asked how much improvement they expected for each item; the following response format was used: 'complete improvement or back to normal', 'a lot of improvement', 'a moderate amount of improvement', 'a little improvement' or 'this expectations does not apply to me/I do not have this expectation' [13]. The total score ranged from 0 to 72, which was recoded into a 100-point scale, with a higher score representing higher expectations. The original English-language survey showed good test-retest reliability and content validity [3,13]. Cronbach's alpha as measure of internal consistency was 0.77 [13].

The Hospital for Special Surgery Knee Replacement Expectations Survey is also developed by Mancuso et al. and consists of 19 items which were constructed by means of interviews with 161 patients [15]. Answers could be given on the same scale as in the Hip Replacement Expectations Survey and scores were recoded into a 100-points scale. The original English-language survey showed good test-retest reliability and content validity; Cronbach's alpha as measure of internal consistency was 0.79 [13,15].

### Translation

The developer of the questionnaires was informed and gave consent to a Dutch translation of the Expectations Surveys (Carol Mancuso, MD, Hospital for Special Surgery, personal communication, 2008).

The Hip Replacement Expectations Survey and Knee Replacement Expectations Survey were translated according to the international guidelines described by Beaton et al. [16]. This method recognises 5 stages: (1) translation, (2) synthesis, (3) back translation, (4) expert committee review and (5) pre-testing. Two persons who had Dutch as their mother tongue and were fluent in English, one informed about the goal and one uninformed, independently translated the questionnaire into Dutch (stage 1). At stage 2 a synthesis was made of these two translations by the two translators of stage 1. Back translation (stage 3) was done independently by two native English speakers fluent in Dutch, one with a medical background and one without, both neither aware nor informed of the concept explored. The expert committee consisting of two translators from stages 1 and 3 and a human movement scientist/epidemiologist (first author) drafted the final version (stage 4), which was pre-tested by interviewing patients after completing the questionnaire. For final versions see Additional files 1 and 2.

### Patients and procedure

Patients on the waiting list for primary total hip or knee arthroplasty at University Medical Center Groningen (UMCG) or Martini Hospital Groningen (MZH) in the Netherlands were sent the Dutch Hip Replacement Expectations Survey or the Dutch Knee Replacement Expectations Survey. In total 112 patients were on the waiting list for a total hip arthroplasty (81 MZH, 31 UMCG) and 101 patients on the waiting list for total knee arthroplasty (76 MZH, 25 UMCG) at the time of the study. They were asked about their age, gender, height and weight (BMI), educational level and living situation. The aim of the study was clarified in the accompanying letter, and it was explained that return of the questionnaire was taken as consent to participate. The study was conducted according to the regulations of the Medical Ethical Committees of both participating hospitals.

To determine test-retest reliability, the surveys were sent again after a two-week interval. This period can be considered short enough to prevent large changes in expectations, and long enough to prevent patients from filling in the questionnaire by memory.

To determine concurrent validity, patients were asked in the first mailing to additionally complete the Expectation WOMAC [17]. The Western Ontario and McMaster Universities Osteoarthritis index (WOMAC) is a frequently-used and recommended disease-specific questionnaire that is found reliable and valid to determine self-report outcome after hip and knee replacement [18,19]. The WOMAC consists of 24 items on pain, stiffness and functional limitations. To determine expectations, the initial wording of the questions was slightly changed: instead of asking how much pain or stiffness and how many limitations patients are experiencing currently, the expectation WOMAC asked the patients how they expect to feel six months after the surgery [17]. Answers could be given on the same 5 point Likert scale as in the original WOMAC, ranging from 'none' to 'extreme'.

### Statistical analyses

Means and standard deviations were calculated for patient characteristics and total scores on the questionnaires. Only complete questionnaires were included in the analyses. To determine test-retest reliability, Intraclass Correlation Coefficients (ICCs) (two-way mixed effects model, absolute agreement) were calculated between total scores of the first and second measurements as well as between the scores on the individual items [20]. An ICC of 0.80 or higher was considered high, as set by Nunnally and Bernstein [21]. Additionally, to determine agreement, Bland and Altman plots were made; in these plots the mean difference (d) between the first and second measurements with corresponding 95% CI and the 95% Limits Of Agreement (LOA) were presented (d ± t_n-1 _× SD_d_) [22]. Cronbach's alphas were determined to assess internal consistency. To determine concurrent validity, Pearson's correlation coefficients were calculated between the total score on the Dutch Hip/Knee Replacement Expectations Survey (first measurement) and the Expectation WOMAC. Moreover, Bland and Altman analyses were performed to determine whether bias occurred. All analyses were done with SPSS 16.0 (SPSS Inc, Chicago).

## Results

### Dutch Hip Replacement Expectations Survey

Of the 112 patients on the waiting list for a total hip arthroplasty 93 patients (83%) returned the Dutch Hip Replacement Expectations Survey and the Expectation WOMAC. These 93 patients received the Dutch Hip Replacement Expectations Survey a second time, and 78 (84%) returned this questionnaire, at a mean of 10.8 days after return of the first questionnaire. Due to missing data, the data of 72 patients were included in the test-retest reliability analysis and data of 86 patients in the validity analysis. The patient characteristics and outcome scores on the first and second assessment of the Dutch Hip Replacement Expectations Survey and the Expectation WOMAC are presented in Table 1.

**Table 1:** Patient characteristics and mean total scores on questionnaires for the reliability and validity study.

	Reliability study		Validity study	
	
	Hip	Knee	Hip	Knee
N	72	46	86	57
Female (N, %)	56 (77.8)	24 (52.2)	63 (73.3)	32 (56.1)
Age in years (mean, SD)	67.5 (9.7)	69.9 (8.2)	67.7 (10.4)	69.6 (8.2)
BMI in kg/m^2 ^(mean, SD)	25.9 (4.0)	27.7 (4.6)	26.1 (5.4)	28.6 (5.1)
Living situation				
Living alone (N, %)	20 (27.8)	14 (30.4)	22 (25.6)	17 (29.8)
Living with partner and/or children (N, %)	52 (72.2)	32 (69.6)	64 (74.4)	40 (70.2)
Highest educational level				
Primary school (N, %)	21 (29.2)	24 (52.2)	27 (31.4)	31 (54.4)
Higher education (N, %)	51 (70.8)	22 (47.8)	59 (68.6)	26 (45.6)
Operation performed in:				
University Medical Center (N, %)	13 (18.1)	7 (15.2)	20 (23.3)	9 (15.8)
General hospital (N, %)	59 (81.9)	39 (84.8)	66 (76.7)	48 (84.2)
Expectation Score A (mean, SD)	66.8 (18.0)	60.4 (20.3)	65.5 (19.8)	61.3 (20.5)
Expectation Score B (mean, SD)	67.3 (19.8)	61.8 (20.8)	NA	NA
Expectation WOMAC Score (mean, SD)	NA	NA	81.1 (15.8)	78.2 (16.6)

NOTE. BMI: Body Mass Index; Expectation Score A: score from first assessment of the Dutch Hip/Knee Replacement Expectations Survey; Expectation Score B: score from second assessment of the Dutch Hip/Knee Replacement Expectations Survey.

**Figure 1:**
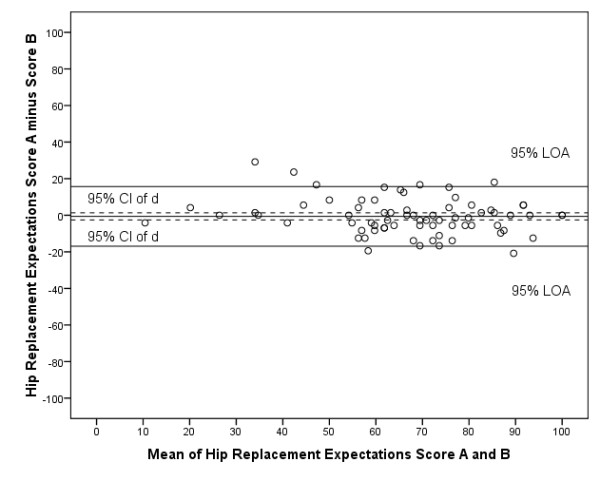
**Bland and Altman plot reliability Dutch Hip Replacement Expectations Survey**. Expectations Score A: score from first assessment of the Dutch Hip/Knee Replacement Expectations Survey; Expectations Score B: score from second assessment of the Dutch Hip/Knee Replacement Expectations Survey; CI: confidence interval; d: mean difference between first and second assessment of the survey; LOA: limits of agreement.

As for agreement, the Bland and Altman plot shows that zero lies within the 95% CI of the mean difference (d) between the first and second measurement of the Dutch Hip Replacement Expectations Survey, indicating no bias (Figure 1). The 95% LOA are -0.6 ± 19.6. The intraclass correlation coefficient between the Hip Replacement Expectations Score of the first and second assessment was 0.87 (95% CI 0.79-0.91). The ICCs of the individual items ranged from 0.52 (item 14) to 0.83 (items 12 and 17) (Table 2). Cronbach's alpha as measure of internal consistency was 0.86 for the Dutch Hip Replacement Expectations Survey (first assessment).

**Table 2:** Intraclass correlation coefficients between the first and second assessments of the Dutch Hip Replacement Expectations Survey, for the total score and the individual items separately.

	ICC	95% CI
**Hip Replacement Expectations Score**	**0.87**	**0.79 - 0.91**
1. Relief of daytime pain	0.71	0.58 - 0.81
2. Relief of pain that interferes with sleep	0.67	0.52 - 0.78
3. Improve ability to walk	0.61	0.44 - 0.74
4. Improve ability to stand	0.72	0.59 - 0.82
5. Get rid of limp	0.76	0.65 - 0.85
6. Remove need for a cane or other assistive device	0.78	0.67 - 0.85
7. Improve ability to climb stairs	0.59	0.41 - 0.72
8. Improve ability to get in or out of a bed, chair or car	0.69	0.55 - 0.79
9. Improve ability to perform daily activities around the home	0.70	0.55 - 0.80
10. Improve ability to perform daily activities away from the home	0.56	0.38 - 0.70
11. Eliminate need for medications	0.55	0.36 - 0.69
12. Be employed for monetary reimbursement	0.83	0.74 - 0.89
13. Improve sexual activity	0.71	0.57 - 0.81
14. Improve ability to exercise or participate in sports	0.52	0.33 - 0.67
15. Improve ability to participate in social activities or recreation	0.60	0.43 - 0.73
16. Improve ability to put on shoes and socks	0.71	0.57 - 0.81
17. Improve ability to cut toenails	0.83	0.74 - 0.89
18. Improve psychological well-being	0.56	0.38 - 0.70

NOTE. ICC: Intraclass Correlation Coefficient; CI: Confidence Interval

**Figure 2:**
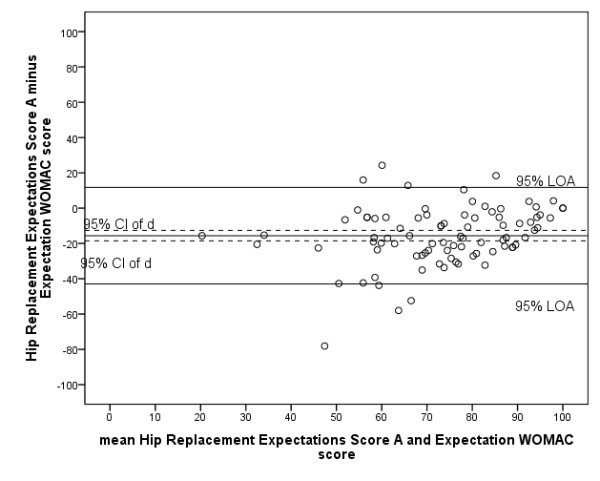
****Bland and Altman plot concurrent validity Dutch Hip Replacement Expectations Survey**. **Expectations Score A: score from first assessment of the Dutch Hip/Knee Replacement Expectations Survey; Expectations Score B: score from second assessment of the Dutch Hip/Knee Replacement Expectations Survey; CI: confidence interval; d: mean difference between first and second assessment of the survey; LOA: limits of agreement.

Regarding concurrent validity, the Pearson's correlation coefficient between the Hip Replacement Expectations Score (first assessment) and the Expectation WOMAC total score was 0.59. The Bland and Altman plot shows that the LOA are -15.6 ± 32.8 (Figure 2). The mean Expectation WOMAC total score was 15.6 points lower than the mean Hip Replacement Expectations Score and zero was not in the 95% CI of d indicating systematic bias.

### Dutch Knee Replacement Expectations Survey

Of the 101 patients on the waiting list for total knee arthroplasty, 65 patients (64%) returned the Dutch Knee Replacement Expectations Survey and the Expectation WOMAC the first time. Of these patients, 54 (83%) returned the Dutch Knee Replacement Expectation Survey that was sent a second time, at a mean of 11.6 days after return of the first questionnaire. Due to missing data, the data of 46 patients were used in the test-retest reliability analysis and the data of 57 patients in the validity analysis. Table 1 shows the patient characteristics and mean outcome scores of the Dutch Knee Replacement Expectations Survey and the Expectation WOMAC.

**Figure 3:**
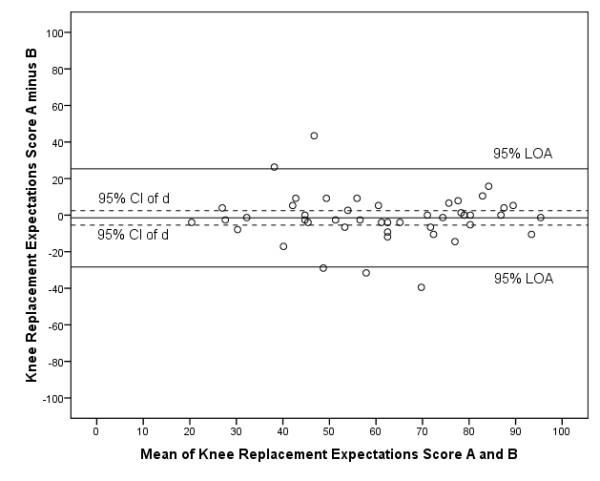
**Bland and Altman plot reliability Dutch Knee Replacement Expectations Survey.** Expectations Score A: score from first assessment of the Dutch Hip/Knee Replacement Expectations Survey; Expectations Score B: score from second assessment of the Dutch Hip/Knee Replacement Expectations Survey; CI: confidence interval; d: mean difference between first and second assessment of the survey; LOA: limits of agreement.

Figure 3 shows the Bland and Altman plot to determine agreement. Zero lies within the 95% CI of the mean difference (d) between the first and second assessment of the Dutch Knee Replacement Expectations Survey, indicating no systematic bias. The 95% LOA were -1.5 ± 26.7. The ICC between the Knee Replacement Expectations Score of the first and second assessment was 0.79 (95% CI 0.66-0.88). The ICCs of the individual items ranged from 0.44 (item 16) to 0.75 (item 14) (Table 3). Internal consistency, as determined with the Cronbach's alpha was 0.91 for the Dutch Knee Replacement Expectations Survey (first assessment).

**Table 3:** Intraclass correlation coefficients between the first and second assessments of the Dutch Knee Replacement Expectations Survey, for the total score and the individual items separately.

	ICC	95% CI
**Knee Replacement Expectations Score**	**0.79**	**0.66 - 0.88**
1. Relief pain	0.74	0.57 - 0.85
2. Improve ability to walk short distance	0.51	0.26 - 0.70
3. Improve ability to walk medium distance	0.70	0.52 - 0.82
4. Improve ability to walk long distance	0.66	0.47 - 0.80
5. Remove the need for a cane, crutch or walker	0.64	0.43 - 0.78
6. Make knee or leg straight	0.63	0.41 - 0.77
7. Improve ability to go up stairs	0.47	0.21 - 0.67
8. Improve ability to go down stairs	0.51	0.26 - 0.70
9. Improve ability to kneel	0.61	0.38 - 0.76
10. Improve ability to squat	0.60	0.37 - 0.76
11. Improve ability to use public transportation, drive	0.70	0.52 - 0.82
12. Be employed for monetary reimbursement	0.68	0.49 - 0.81
13. Improve ability to participate in recreation	0.73	0.55 - 0.84
14. Improve ability to perform daily activities	0.75	0.58 - 0.85
15. Improve ability to exercise or participate in sports	0.74	0.57 - 0.85
16. Improve ability to change position	0.44	0.18 - 0.65
17. Improve ability to interact with others	0.59	0.36 - 0.75
18. Improve sexual activity	0.71	0.53 - 0.83
19. Improve psychological well-being	0.68	0.48 - 0.81

NOTE. ICC: Intraclass Correlation Coefficient; CI: Confidence Interval

**Figure 4:**
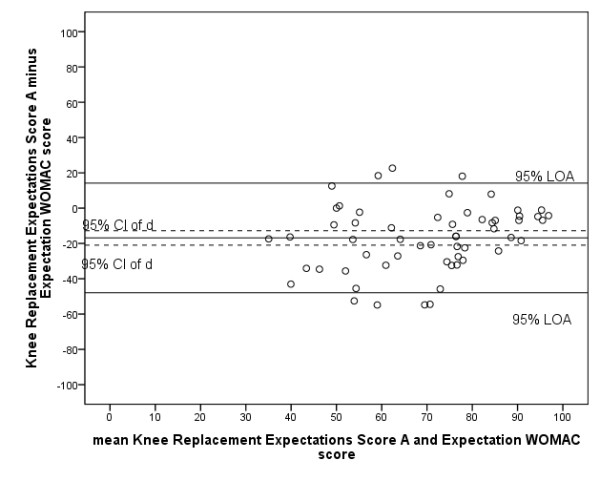
**Bland and Altman plot concurrent validity Dutch Knee Replacement Expectations Survey**. Expectations Score A: score from first assessment of the Dutch Hip/Knee Replacement Expectations Survey; Expectations Score B: score from second assessment of the Dutch Hip/Knee Replacement Expectations Survey; CI: confidence interval; d: mean difference between first and second assessment of the survey; LOA: limits of agreement.

To determine concurrent validity, the Pearson's correlation coefficient between the Knee Replacement Expectations Score (first assessment) and the Expectation WOMAC total score was determined, which was 0.52. 95% LOA were -16.9 ± 37.2 (Figure 4). As the mean Expectation WOMAC score was 16.9 points lower than the mean Knee Replacement Expectations Score and significantly different from zero, systematic bias was present.

## Discussion

As no questionnaires are available in the Dutch language to determine preoperative expectations of patients on a waiting list for total hip or knee arthroplasty, the first aim of this study was to translate the English-language Hospital for Special Surgery Hip Replacement Expectations Survey and the Hospital for Special Surgery Knee Replacement Expectations Survey [3,10,13,15]. The surveys were translated according to the method described by Beaton et al., which is the official method according to the American Association of Orthopaedic Surgeons (AAOS) [16].

Second aim of the study was to determine the test-retest reliability and concurrent validity of the Dutch Hip Replacement Expectations Survey and the Dutch Knee Replacement Expectations Survey. The results of the reliability study show that both Dutch surveys have good test-retest reliability and internal consistency. The Bland and Altman analyses indicated no bias between the first and second measurements. The Intraclass Correlation Coefficients for the total scores were close to or above the criterion of 0.80 of Nunnally and Bernstein [21], and can therefore be considered high (0.79 and 0.87 for the Knee and Hip Survey, respectively). The item that scored the lowest ICC in the Dutch Hip Replacement Expectations Survey was the expectation of the ability to exercise or participate in sports. By contrast, this item scored high in the Dutch Knee Replacement Expectations Survey. When looking at the individual items of the Dutch Knee Replacement Expectations Survey, the lowest ICCs were found for the expectation regarding the ability to change position and the ability to climb stairs. This latter item also had a moderate ICC in the Dutch Hip Replacement Expectations Survey. It can only be speculated why patients rate their expectations differently when assessed twice. One reason might be that patients find it hard to estimate a certain expectation, resulting in a different answer at the second assessment. Overall, all ICCs were moderate to high (between 0.44 and 0.83) and the differences between the items are small, indicating good test-retest reliability of both surveys. Compared to the original English-language surveys, the internal consistency as determined with Cronbach's alpha was higher in the Dutch-language surveys (Hip 0.86 vs 0.77 in the original version; Knee 0.91 vs 0.79 in the original version) [13]. Both values satisfied the minimum criterion of 0.80 set by Nunnally and Bernstein [21]. No additional data is available concerning the reliability of the original surveys, therefore further comparison with the English-language version is not possible.

As there is no instrument available to determine patient expectations which can be considered the gold standard, the only available questionnaire described in the literature, the Expectation WOMAC, was chosen to determine concurrent validity of the Dutch Hip Replacement Expectations Survey and the Dutch Knee Replacement Expectations Survey [17]. Although the Dutch WOMAC is considered reliable and valid, and is only slightly adapted to result in the Expectation WOMAC, the psychometric properties of the Expectation WOMAC are unknown. To determine concurrent validity, the Pearson's correlations are calculated between the two Dutch Expectations Surveys and the Expectation WOMAC, which were moderate; a correlation between 0.4 and 0.6 is evidence that the same construct is being embraced [23]. However, the Bland and Altman analyses showed considerable bias between the two measures; the mean Expectation WOMAC score was systematically over 15 points lower than the mean score on the Dutch Hip/Knee Replacement Expectation Surveys. It is our hypothesis that the way the Expectation WOMAC is adapted from the original WOMAC results in answers whereby the patients also considers the current status. The bias therefore might reflect a poor validity of the Expectation WOMAC rather than of the Dutch Hip/Knee Replacement Expectations Surveys. An alternative way to determine validity would be using the expectations of orthopaedic surgeons as reference, however this is questionable considering the differences that exist between the expectations of patients and those of orthopaedic surgeons [9].

One of the strengths of the current study is that participants were patients from a university as well as a general hospital. The study also had some limitations. First, not all patients were willing to participate in the study. The response rate of the first mailing was 83% in the hip replacement group and 64% in the knee replacement group. When responders and non-responders represent different patients groups, results of the study might not be generalizable to all total hip and knee replacement patients. Second, some questionnaires had to be excluded from the analyses due to missing values. This is inherent to this older patient group, who is often unfamiliar with filling in questionnaires. One way to avoid missing values is to let patients complete the questionnaires in the hospital. Although the patients are more likely to give socially desirable answers, the questionnaire can be checked for missing values when turned in.

Until now, research on patients' expectations before total hip and knee replacement is scarce and has to our knowledge only been performed in the United States, United Kingdom, Australia and Canada, with only one study comparing three different (English-language) countries [24]. Now that the Hip Replacement Expectations Survey and the Knee Replacement Expectations Survey are available in Dutch, it is possible to determine patients' expectations of total hip or knee replacement in another culture and healthcare setting. The surveys can be used to guide preoperative discussions about expectations between patients and physicians in the outpatient clinic, and in preoperative education classes aiming to change unrealistic expectations. Moreover, cross-cultural comparison is possible and an important future research topic.

## Conclusions

In conclusion, the Dutch Hip Replacement Expectations Survey and the Dutch Knee Replacement Expectations Survey are both reliable instruments to determine patient expectations before total hip or knee replacement. With respect to concurrent validity it can be concluded that the correlation between both Surveys and the Expectation WOMAC was moderate confirming that the same construct was determined. However, a systematic bias was found; patients scored systematically lower on the Expectation WOMAC compared to the Dutch Expectations Surveys.

## List of abbreviations

WOMAC: Western Ontario and McMaster Universities Osteoarthritis index; BMI: Body Mass Index; ICC: Intraclass Correlation Coefficient; CI: Confidence Interval; MZH: Martini Hospital Groningen; UMCG: University Medical Center Groningen.

## Competing interests

The authors declare that they have no competing interests.

## Authors' contributions

IAS originated the idea for the study, set up the design of the study, coordinated the study, performed the data analysis and drafted the manuscript. IR, WZ and MS participated in the design of the study and were involved in drafting the manuscript. IR provided statistical consultation. JR and SB contributed to the conception of the study, supervised the project and revised the manuscript critically. All authors have read and approved the final manuscript.

## Pre-publication history

The pre-publication history for this paper can be accessed here:

http://www.biomedcentral.com/1471-2474/11/242/prepub

## Supplementary Material

Additional file 1 **Dutch Hip Replacement Expectations Survey**. Click here for file

Additional file 2 **Dutch Knee Replacement Expectations Survey**. Click here for file
