# Supplementary material for: Reliability and concurrent validity of the Dutch hip and knee replacement expectations surveys
Source: BMC Musculoskelet Disord. 2010 Oct 19;11:242. doi: 10.1186/1471-2474-11-242 (PMC2973929; doi:10.1186/1471-2474-11-242)
Supplement: Additional file 1 — Dutch Hip Replacement Expectations Survey. [file 1471-2474-11-242-S1.PDF]

Vragenlijst Verwachtingen van een Totale Knievervanging

Wilt u alstublieft het nummer omcirkelen dat uw antwoord op de vraag het beste omschrijft.

Hoeveel verlichting of verbetering verwacht u op de volgende gebieden als gevolg van uw totale knievervanging?

	**Terug naar normaal** of totale verbetering	**Niet terug naar normaal, maar …**			Ik heb deze verwachting niet of deze verwachting is niet op mij van toepassing
		Veel verbetering	Middelmatige verbetering	Een kleine verbetering	
Verlichting van pijn	1	2	3	4	5
Verbeteren van het loopvermogen op:<br>** korte afstanden (binnenshuis, een huizenblok)	1	2	3	4	5
** middellange afstanden (een stukje lopen, tot 1,5 km)	1	2	3	4	5
** lange afstanden (meer dan 1,5 km)	1	2	3	4	5
Het niet meer nodig hebben van een stok, kruk of rollator	1	2	3	4	5
Het strekken van knie of been	1	2	3	4	5
Verbeteren van het vermogen trappen op te gaan	1	2	3	4	5
Verbeteren van het vermogen trappen af te gaan	1	2	3	4	5
Verbeteren van het vermogen om te knielen	1	2	3	4	5
Verbeteren van het vermogen te hurken	1	2	3	4	5
Verbeteren van het vermogen om van het openbaar vervoer gebruik te maken of te rijden	1	2	3	4	5
Betaald werk kunnen doen	1	2	3	4	5
Verbeteren van het vermogen deel te nemen aan recreatieve activiteiten (bijvoorbeeld dansen, plezierreisjes)	1	2	3	4	5
Verbeteren van het vermogen dagelijkse activiteiten uit te voeren (bijvoorbeeld huishoudelijke werkzaamheden, dagelijkse routine)	1	2	3	4	5
Verbeteren van het vermogen lichamelijk actief te zijn of deel te nemen aan sport	1	2	3	4	5
Verbeteren van het vermogen om van positie te veranderen (bijvoorbeeld van zitten naar staan of van staan naar zitten)	1	2	3	4	5
Verbeteren van het vermogen om te gaan met anderen (bijvoorbeeld voor iemand zorgen, spelen met kinderen)	1	2	3	4	5
Verbeteren van seksuele activiteit	1	2	3	4	5
Verbeteren van psychologisch welzijn	1	2	3	4	5

■ Vertaling van vragenlijst gemaakt door het Hospital for Special Surgery (New York, NY, USA).
Nederlandse versie door Van den Akker-Scheek et al. 2008. Universitair Medisch Centrum Groningen.
