# Supplementary material for: Reliability and concurrent validity of the Dutch hip and knee replacement expectations surveys
Source: BMC Musculoskelet Disord. 2010 Oct 19;11:242. doi: 10.1186/1471-2474-11-242 (PMC2973929; doi:10.1186/1471-2474-11-242)
Supplement: Additional file 2 — Dutch Knee Replacement Expectations Survey. [file 1471-2474-11-242-S2.PDF]

Vragenlijst Verwachtingen van een Totale Heupvervanging

Wilt u alstublieft het nummer omcirkelen dat uw antwoord op de vraag het beste omschrijft.

Hoeveel verlichting of verbetering verwacht u op de volgende gebieden als gevolg van uw totale heupvervanging?

	**Terug naar normaal** of totale verbetering	**Niet terug naar normaal, maar …**			Ik heb deze verwachting niet of deze verwachting is niet op mij van toepassing
		Veel verbetering	Middelmatige verbetering	Een kleine verbetering	
Verlichting van pijn die overdag optreedt	1	2	3	4	5
Verlichting van pijn tijdens slapen	1	2	3	4	5
Verbeteren van het loopvermogen	1	2	3	4	5
Verbeteren van het vermogen te staan	1	2	3	4	5
Niet langer mank lopen	1	2	3	4	5
Het niet meer nodig hebben van een stok of andere hulpmiddelen	1	2	3	4	5
Verbeteren van het vermogen trappen op te lopen	1	2	3	4	5
Verbeteren van het vermogen in of uit bed, stoel of auto te komen	1	2	3	4	5
Verbeteren van het vermogen dagelijkse activiteiten rond het huis te verrichten (bijvoorbeeld, huishoudelijke klusjes, tuinieren)	1	2	3	4	5
Verbeteren van het vermogen dagelijkse activiteiten buiten het huis te verrichten (bijvoorbeeld, winkelen, vrijwilligerswerk)	1	2	3	4	5
Het niet langer nodig hebben van medicijnen	1	2	3	4	5
Betaald werk kunnen doen	1	2	3	4	5
Verbeteren van seksuele activiteit	1	2	3	4	5
Verbeteren van het vermogen lichamelijk actief te zijn of deel te nemen aan sport	1	2	3	4	5
Verbeteren van het vermogen deel te nemen aan sociale of recreatieve activiteiten	1	2	3	4	5
Verbeteren van het vermogen schoenen en sokken aan te trekken	1	2	3	4	5
Verbeteren van het vermogen teennagels te knippen	1	2	3	4	5
Verbeteren van psychologisch welzijn	1	2	3	4	5

■ Vertaling van vragenlijst gemaakt door het Hospital for Special Surgery (New York, NY, USA).
Nederlandse versie door Van den Akker-Scheek et al. 2008. Universitair Medisch Centrum Groningen.
